# Prevalence of asymptomatic cytomegalovirus (CMV) infection in newborns in northeast Florida

**DOI:** 10.3389/fepid.2023.1270374

**Published:** 2024-01-03

**Authors:** Rana Alissa, Nizar Maraqa, Patty D. Williams, Jennifer A. Hipp, Sfurti Nath, Nicole S. Torres, Tiffany Lee, Amr Matoq, Mobeen Rathore

**Affiliations:** ^1^ Department of Pediatrics, College of Medicine - Jacksonville, University of Florida, Jacksonville, FL, United States; ^2^ Department of Pediatrics, University of Miami Health System, Miami, FL, United States

**Keywords:** congenital CMV, sensorineural hearing loss (SNHL), universal screening, early intervention, urine testing, saliva testing

## Abstract

**Background:**

Congenital cytomegalovirus (CMV) infection is the leading cause of hearing loss and neurocognitive delay among children. Affected infants may be asymptomatic at birth and even pass their universal hearing screen. Early identification of CMV-infected infants will allow earlier detection, evaluation and management. The prevalence of congenital CMV infection in the developed world varies geographically from 0.6% to 0.7% of all deliveries and certain regions are at higher risk. The prevalence of congenital CMV is unknown for our region.

**Aim:**

The purpose of this study was to determine the prevalence of CMV infection among the neonatal population at an urban, tertiary hospital in northeast Florida which serves a large population of patients with low socioeconomic status to assess if universal screening program for congenital asymptomatic CMV infection can be determined.

**Methods:**

The study was submitted and approved by our Institutional Review Board. We tested the urine for CMV infection in 100 asymptomatic newborns (>32 weeks gestational age and >1,750 g weight at the time of delivery) delivered between June 2016 and July 2017.

**Results:**

Urine CMV was tested on 100 infants. One infant had a positive urine NAAT for CMV, making the prevalence of congenital CMV infection among asymptomatic newborns in our hospitals' population 1%.

**Conclusion:**

CMV prevalence in our setting of an urban, tertiary hospital is relatively consistent with the national average of all congenital CMV infections. A policy of universal screening for congenital CMV may be necessary.

## Background and significance

Congenital cytomegalovirus (CMV) infection is the leading cause of non-hereditary sensorineural hearing loss (SNHL) (1–4), and the most frequent known viral cause of neurodevelopmental delay (5). Affected infants may be asymptomatic at birth and even pass their universal hearing screen (3). It is estimated that CMV affects 0.6%–0.7% of live births in industrialized countries (6–8). In the United States, where the annual birth cohort is approximately 4 million, between 20,000 and 40,000 babies are estimated to be born each year with congenital CMV infection (6). Among the congenitally infected neonates, 10%–15% have CMV specific symptoms at delivery, of whom 40%–58% will have long-term sequalae including SNHL, neurologic deficits, developmental delay and death in the newborn period (6–10). SNHL occurs at a lower rate (2%) among the congenitally infected neonates who are asymptomatic at birth (11). However, because there are more asymptomatic neonates than symptomatic ones, the majority of cases of SNHL caused by CMV occur in the asymptomatic group (1, 7). Overall, although congenital CMV is a rare infection, it accounts for 10% of hearing loss at birth and 35% of moderate-to-severe late-onset hearing loss (1, 12).

Newborn screening for CMV infection will identify infants at-risk for congenital CMV infection early for timely diagnosis and intervention during crucial periods of speech and language development (13).

Antiviral treatment of neonates with symptomatic congenital CMV disease is now the standard of care (14). Initially, intravenous (IV) ganciclovir administered for 6 weeks to infants with symptomatic congenital CMV disease that involves the central nervous system (CNS) improved audiologic outcomes of those infants at 6 months of age, but there was suggestion that this benefit could wane over the first 2 years of life (15–18). Additionally, a few studies have documented a rebound in CMV viral load following cessation of therapy, but have not followed subjects for longer than one month (15, 18). Further research showed that six months of treatment with oral valganciclovir, a prodrug of ganciclovir, achieved similar plasma concentration to IV ganciclovir (19). This treatment also improved hearing and developmental outcomes in the long term compared with six weeks of IV ganciclovir treatment (20, 21). The American Academy of Pediatrics now recommends six months of oral valganciclovir as standard therapy for infants born with symptomatic congenital CMV disease (22).

Given the benefits demonstrated from longer-term antiviral treatment of infants with symptomatic congenital CMV disease, it is highly probable that infants with asymptomatic congenital CMV infection who are treated with valganciclovir will have protection against hearing deterioration (14). In order to treat infants with potential congenital CMV infection, it becomes necessary to identify those who are infected (23). The only way to identify neonates who are asymptomatically infected with CMV during gestation is through universal screening for CMV infection at birth (24).

The prevalence of CMV infection in women of childbearing age varies depending on the geographical area and is higher at or above poverty level (25). The prevalence of CMV infection among women of childbearing age in northeast Florida is not known. Consequently, the asymptomatic CMV infection prevalence in newborns in northeast Florida is also not known. A study of prevalence of asymptomatic CMV infection in the newborns in northeast Florida will allow us to better understand the epidemiology and benefit of any potential intervention to prevent complications, including SNHL (23).

Our study assessed the prevalence of congenital CMV among asymptomatic newborns at an urban, tertiary hospital in northeast Florida with a large population of patients with low socioeconomic status, Table 1 explains the payors category in our Mother-Baby unit throughout the subjects' enrollment of this study.

**Table 1:** Payor category in the mother-baby unit during the study time.

Payor category	% Total
Charity	9.29
Commercial	0.22
Exchange plans	7.55
Managed care HMO	3.30
Managed care PPO	7.11
Medicaid	7.51
Medicaid HMO	54.79
Medicaid pending	6.64
Medicare	0.29
Medicare HMO	0.25
Self-pay	0.47
Tricare	2.58
Grand total	100

### Specific aims

The purpose of this study was to determine the prevalence of CMV infection among the neonatal population in an urban, tertiary hospital in northeast Florida with a large population of patients with low socioeconomic status so that the feasibility of a universal screening program for congenital asymptomatic CMV infection can be determined.

### Research design and methods

This study was submitted and approved by our Institutional Review Board (IRB).

- Study population:

The study population consisted of male and female infants delivered at our facility whose gestational age was >32 weeks at birth and weighed >1,750 g at the time of enrollment. A signed informed consent form was obtained from agreeable parent(s) or legal guardian(s) by the study team for recruitment, as soon as possible after the infant's birth.

Symptomatic congenital CMV disease, as manifested by one or more of the following signs were excluded from the study:

• Thrombocytopenia (if known)
• Petechiae
• Hepatomegaly
• Splenomegaly
• Small for gestational age (SGA)
• Intrauterine growth restriction
• Hepatitis (elevated transaminases and/or direct bilirubin), if known
• Central nervous system involvement attributable to CMV (such as microcephaly; radiographic abnormalities indicative of CMV CNS disease [if known]; abnormal CSF indices for age [if known]; chorioretinitis, if known; and/or positive CMV PCR from CSF [if known])
• Sensorineural hearing deficits as detected by formal brainstem evoked response (not a screening ABR)
• Imminent demise
• Prior or current treatment with ganciclovir, valganciclovir, foscarnet, cidofovir, brincidofovir, maribivir, or letermovir
• Maternal receipt of CMV hyperimmune globulin during pregnancy
• Breastfeeding from mother who is receiving any of the following medications: ganciclovir, valganciclovir, foscarnet, cidofovir, brincidofovir, maribivir or letermovir

- Study design:

One hundred (100) newborns delivered at UF Health Jacksonville between June 2016 and July 2017 were randomly enrolled and tested for the presence of urine CMV by nucleic acid amplification test (NAAT). Funding for this study was partially provided by a University of Florida College of Medicine- Jacksonville “Dean's Research Fund” grant awarded to the participating pediatric residents.

All infants delivered in our facility were screened by the study team for eligibility. The new mothers of all the eligible subjects were approached by the study team shortly after the time of delivery and when agreeable, they signed an informed consent form.

Urine was collected from participating newborns using a urine collection bag and the specimen was transferred to the hospital's laboratory for testing according to laboratory protocols and test manufacturer recommendations.

The newborns who were found to have CMV infection were evaluated for sensorineural hearing loss and managed per the current standard of care.

We reviewed the electronic health records of the enrolled newborns for collection of demographics and pertinent maternal and perinatal history, examination and laboratory data (including gender, ethnicity, mode of delivery, birth weight, hearing screen results, maternal age, parity, residence zip code, and results of prenatal infectious screening tests).

## Results

Urine was successfully collected from 100 of 104 recruited infants. Four infants without a urine sample were excluded from further analysis. All the CMV tests were performed within the first three days of the participating newborns' life. One infant had a positive urine NAAT for CMV making the prevalence of congenital CMV infection among asymptomatic newborns in our geographic location 1%. The CMV infected newborn was delivered at 41 weeks gestational age (GA) via spontaneous vaginal delivery to a 16-year-old primigravida woman who was incarcerated at the time of delivery. The mother was also positive for group B *Streptococcus* (GBS) and chlamydia.

Our population of 100 newborns included 57 (57%) females. The majority were African American (56%), followed by Caucasian (21%), other (18%) and unknown (5%). There were 77 vaginal deliveries and 23 cesarean sections. The average GA was 38/6 weeks (range: 35–43 weeks). The average birth weight was 3,316 grams (range: 2,170–4,620 grams) and there were only 3 infants with birth weights < 2,500 grams. Average head circumference was 33.8 cm (range: 28.5–37 cm). Hearing screening was normal in 97 (97%) infants, 2 failed and 1 was unknown.

**Figure 1:**
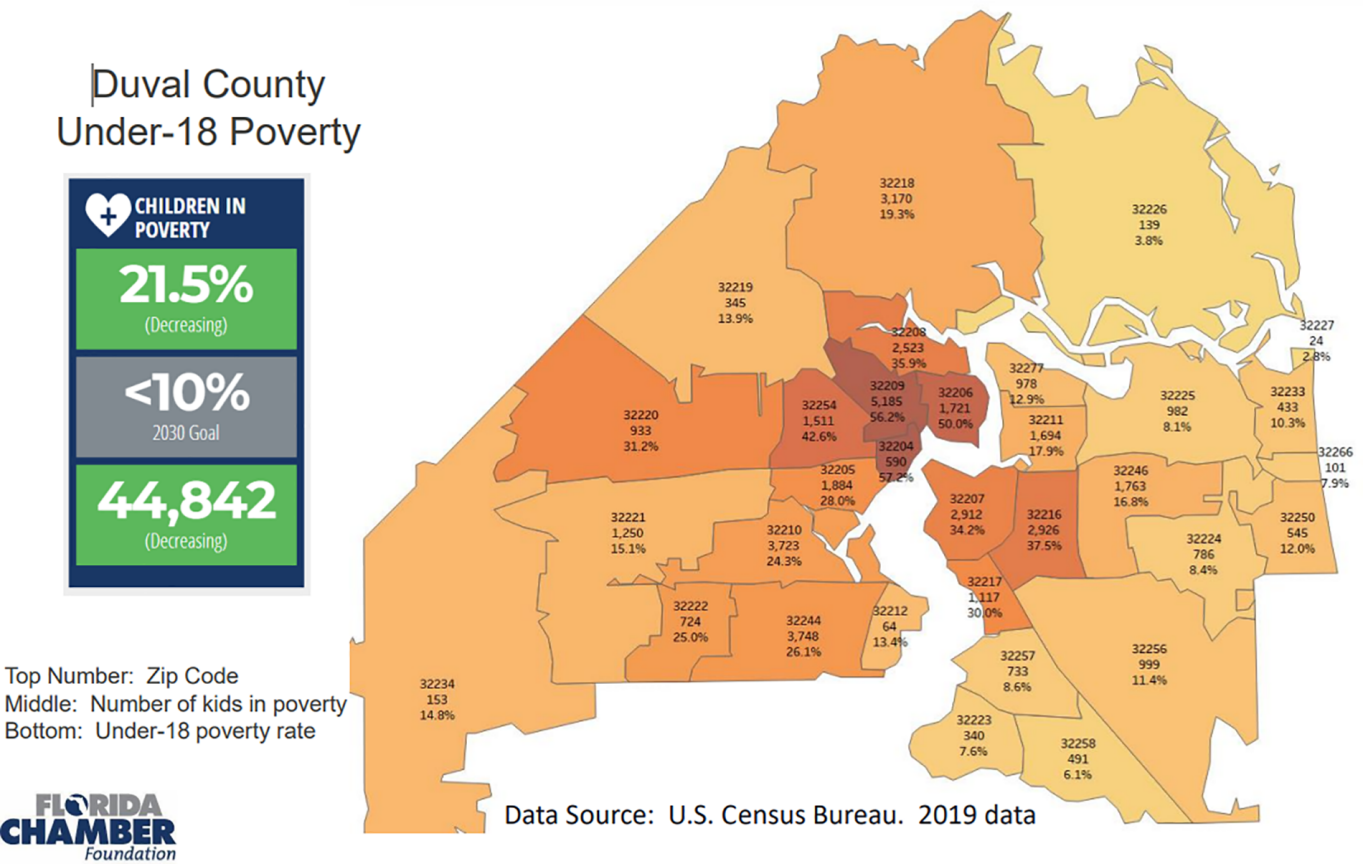
Under-18 poverty rate in the zip codes which represent our hospital's population.

Average maternal age was 26.5 years (range: 16–45 years, 11 were under 20 years; 13 were 35 years or older) and average parity was 3.2 (range: 1–13; 24 primigravida and 19 gravida 5 or greater). With regard to maternal prenatal infectious screening tests; 35 (35%) mothers were GBS positive, 3 (3%) had reactive RPR, 1 (1%) was positive for chlamydia, and 16 (16%) had an unknown gonorrhea and/or chlamydia status. As far as maternal residence, the mothers lived in 31 different zip code areas with 20 (20%) living in the same zip code as the hospital (32209) which has an Under-18 Poverty Rate of 56.2%. The majority of the rest lived in zip codes with an Under-18 Poverty Rates of 35.9–57.2 (Figure 1). Our infant lived in one of these zip codes with a high Under-18 Poverty Rate.

Our infant passed both the otoacoustic emissions testing (OAE) at birth and the auditory brainstem response (ABR) test. His head US, eye exam, liver enzymes and complete blood counts were within normal limits. He did not require any medication treatment or any further intervention.

## Discussion

This study screened asymptomatic infants born in our hospital between June 2016 and July 2017 and it resulted in 1 (1%) congenital CMV infection. Fortunately, all the investigations for the infant with positive congenital CMV were reassuring and no interventions were required. The fact that the CMV prevalence in our hospital was consistent with the national average despite our small sample size suggests the possibility of missing a significant number of asymptomatic infants with congenital CMV infection.

Children with asymptomatic congenital CMV have a higher rate of SNHL with progression throughout childhood even when the hearing loss was unilateral. For these infants with SNHL because of congenital CMV, ongoing audiological follow up is crucial to receive appropriate and timely interventions (26).

In 2013, the state of Utah passed a law requiring infants who fail their newborn hearing screen to be tested for CMV infection. Other states: Connecticut, Florida, Iowa, Kentucky, New York, Utah, and Virginia passed the same law later on. This type of “targeted screening” for congenital CMV infection will not identify the overwhelming majority of at-risk neonates, since only 10%–15% of asymptomatic congenital CMV infants develop CMV-associated SNHL and have hearing loss present at birth or years later (27). Furthermore, newborn hearing screening programs do not detect all CMV-related hearing loss (26). Therefore, the cost-effectiveness of universal screening for congenital CMV infection estimated a 12% reduction in the costs associated with hearing loss due to an early intervention (27). Additionally, universal screening for congenital CMV infection has recently been discussed by a Recommendations Group in the 5th International Congenital Cytomegalovirus conference (28). All the above emphasize the importance of universal CMV screening.

Many rare diseases are included in the universal screening such as Maple syrup urine disease (MSUD) with prevalence of 1 case per 185,000 live births (29), Phenylketonuria (PKU) with prevalence of 1 in 23,930 live births (30). Despite their comparatively low incidence, MSUD and PKU are included in the newborn screening. There are many more examples of much rarer diseases than congenital CMV that are included in the universal newborn screen. “Furthermore, the implementation of the universal newborn hearing screening in 1999 (31), and the screening for Critical Congenital Heart Disease (CCHD) in 2011 in the US (32) as a point-of-care newborn screening, gives a variety of options on managing the universal screening for congenital CMV by sending the urine specimens either to the State lab (similar to MSUD and PKU testing), or processing it in the birthing hospital's lab as a point-of care test (similar to the universal hearing screening and the CCHD).”

In February 8, 2023 Minnesota became the first state to screen all newborns for CMV infection.

A policy of universal CMV screening in the rest of the United States may be warranted with all the data available thus far.

This study's limitations include the small sample size and our inability to enroll infants who were <35 weeks GA and <2,000 g who were admitted to the Neonatal Intensive Care Unit due to administrative reasons despite meeting the inclusion criteria.

## Conclusion

CMV prevalence in our setting of an urban, tertiary hospital in northeast Florida is relatively consistent with the national prevalence. However, further studies regarding screening for congenital CMV in asymptomatic infants with SNHL might be necessary to implement a policy of universal screening.

## Data Availability

The original contributions presented in the study are included in the article/Supplementary Material, further inquiries can be directed to the corresponding author.

## References

[1] Morton CC Nance WE. Newborn hearing screening–a silent revolution. N Engl J Med. (2006) 354(20):2151–64. 10.1056/NEJMra050700 16707752

[2] Fowler KB McCollister FP Dahle AJ Boppana S Britt WJ Pass RF. Progressive and fluctuating sensorineural hearing loss in children with asymptomatic congenital cytomegalovirus infection. J Pediatr. (1997) 130(4):624–30. 10.1016/s0022-3476(97)70248-8 9108862

[3] Fowler KB Dahle AJ Boppana SB Pass RF. Newborn hearing screening: will children with hearing loss caused by congenital cytomegalovirus infection be missed? J Pediatr. (1999) 135(1):60–4. 10.1016/s0022-3476(99)70328-8 10393605

[4] Fowler KB Boppana SB. Congenital cytomegalovirus (CMV) infection and hearing deficit. J Clin Virol. (2006) 35(2):226–31. 10.1016/j.jcv.2005.09.016 16386462

[5] Cheeran MC Lokensgard JR Schleiss MR. Neuropathogenesis of congenital cytomegalovirus infection: disease mechanisms and prospects for intervention. Clin Microbiol Rev. (2009) 22(1):99–126. 10.1128/CMR.00023-08 19136436 PMC2620634

[6] Swanson EC Schleiss MR. Congenital cytomegalovirus infection: new prospects for prevention and therapy. Pediatr Clin North Am. (2013) 60(2):335–49. 10.1016/j.pcl.2012.12.008 23481104 PMC3807860

[7] Dollard SC Grosse SD Ross DS. New estimates of the prevalence of neurological and sensory sequelae and mortality associated with congenital cytomegalovirus infection. Rev Med Virol. (2007) 17(5):355–63. 10.1002/rmv.544 17542052

[8] Manicklal S Emery VC Lazzarotto T Boppana SB Gupta RK. The “silent” global burden of congenital cytomegalovirus. Clin Microbiol Rev. (2013) 26(1):86–102. 10.1128/CMR.00062-12 23297260 PMC3553672

[9] Dahle AJ Fowler KB Wright JD Boppana SB Britt WJ Pass RF. Longitudinal investigation of hearing disorders in children with congenital cytomegalovirus. J Am Acad Audiol. (2000) 11(5):283–90. 10.1055/s-0042-1748054 10821506

[10] Boppana SB Ross SA Fowler KB. Congenital cytomegalovirus infection: clinical outcome. Clin Infect Dis. (2013) 57 Suppl 4(Suppl 4):S178–81. 10.1093/cid/cit629 24257422 PMC4471438

[11] Lanzieri TM Chung W Flores M Blum P Caviness AC Bialek SR Congenital cytomegalovirus longitudinal study group. Hearing loss in children with asymptomatic congenital cytomegalovirus infection. Pediatrics. (2017) 139(3):e20162610. 10.1542/peds.2016-2610 28209771 PMC5330400

[12] Grosse SD Ross DS Dollard SC. Congenital cytomegalovirus (CMV) infection as a cause of permanent bilateral hearing loss: a quantitative assessment. J Clin Virol. (2008) 41(2):57–62. 10.1016/j.jcv.2007.09.004 17959414

[13] Boppana SB Ross SA Shimamura M Palmer AL Ahmed A Michaels MG National institute on deafness and other communication disorders CHIMES study. Saliva polymerase-chain-reaction assay for cytomegalovirus screening in newborns. N Engl J Med. (2011) 364(22):2111–8. 10.1056/NEJMoa1006561 21631323 PMC3153859

[14] Lanzieri TM Pesch MH Grosse SD. Considering antiviral treatment to preserve hearing in congenital CMV. Pediatrics. (2023) 151(2):e2022059895. 10.1542/peds.2022-059895 36695050 PMC10348364

[15] Whitley RJ Waldron KG Honey S Cloud G Harris R Macdonald A Ganciclovir treatment of symptomatic congenital cytomegalovirus infection: results of a phase II study. National institute of allergy and infectious diseases collaborative antiviral study group. J Infect Dis. (1997) 175(5):1080–6. 10.1086/516445 9129069

[16] Trang JM Kidd L Gruber W Storch G Demmler G Jacobs R Linear single-dose pharmacokinetics of ganciclovir in newborns with congenital cytomegalovirus infections. NIAID collaborative antiviral study group. Clin Pharmacol Ther. (1993) 53(1):15–21. 10.1038/clpt.1993.4 8380762

[17] Zhou XJ Gruber W Demmler G Jacobs R Reuman P Adler S Population pharmacokinetics of ganciclovir in newborns with congenital cytomegalovirus infections. NIAID collaborative antiviral study group. Antimicrob Agents Chemother. (1996) 40(9):2202–5. 10.1128/AAC.40.9.2202 8878608 PMC163500

[18] Kimberlin DW Lin CY Sánchez PJ Demmler GJ Dankner W Shelton M Effect of ganciclovir therapy on hearing in symptomatic congenital cytomegalovirus disease involving the central nervous system: a randomized, controlled trial. J Pediatr. (2003) 143(1):16–25. 10.1016/s0022-3476(03)00192-6 12915819

[19] Kimberlin DW Acosta EP Sánchez PJ Sood S Agrawal V Homans J Pharmacokinetic and pharmacodynamic assessment of oral valganciclovir in the treatment of symptomatic congenital cytomegalovirus disease. J Infect Dis. (2008) 197(6):836–45. 10.1086/528376 18279073

[20] Kimberlin DW Jester PM Sánchez PJ Ahmed A Boger RA Michaels MG Valganciclovir for symptomatic congenital cytomegalovirus disease. N Engl J Med. (2015) 372(10):933–43. 10.1056/NEJMoa1404599 25738669 PMC4401811

[21] American Academy of Pediatrics. Cytomegalovirus infection. In: Kimberlin DW Brady MT Jackson MA Long SS, editors. Red Book: 2015 Report of the Committee on Infectious Diseases. 30th ed. Elk Grove Village, IL: American Academy of Pediatrics (2015). p. 317–22.

[22] http://www.gene.com/download/pdf/valcyte_prescribing.pdf.

[23] Fowler KB. Congenital cytomegalovirus infection: audiologic outcome. Clin Infect Dis. (2013) 57 Suppl 4(Suppl 4):S182–4. 10.1093/cid/cit609 24257423 PMC3836573

[24] Chiereghin A Pavia C Turello G Borgatti EC Pillastrini FB Gabrielli L Universal newborn screening for congenital cytomegalovirus infection—from infant to maternal infection: a prospective multicenter study. Front Pediatr. (2022) 10:909646. 10.3389/fped.2022.909646 35874574 PMC9298552

[25] Fowler K Mucha J Neumann M Lewandowski W Kaczanowska M Grys M A systematic literature review of the global seroprevalence of cytomegalovirus: possible implications for treatment, screening, and vaccine development. BMC Public Health. (2022) 22(1):1659. 10.1186/s12889-022-13971-7 36050659 PMC9435408

[26] Centers for Disease Control and Prevention. U.S. Public health impact of congenital cytomegalovirus infection. MMWR—CDC Surveillance Summaries. (1992) 41:35–9.

[27] Gantt S Dionne F Kozak FK Goshen O Goldfarb DM Park AH Cost-effectiveness of universal and targeted newborn screening for congenital cytomegalovirus infection. JAMA Pediatr. (2016) 170(12):1173–80. 10.1001/jamapediatrics.2016.2016 27723885

[28] Rawlinson WD Boppana SB Fowler KB Kimberlin DW Lazzarotto T Alain S Congenital cytomegalovirus infection in pregnancy and the neonate: consensus recommendations for prevention, diagnosis, and therapy. Lancet Infect Dis. (2017) 17(6):e177–88. 10.1016/S1473-3099(17)30143-3 28291720

[29] Edelmann L Wasserstein MP Kornreich R Sansaricq C Snyderman SE Diaz GA. Maple syrup urine disease: identification and carrier-frequency determination of a novel founder mutation in the ashkenazi Jewish population. Am J Hum Genet. (2001) 69(4):863–8. 10.1086/323677 11509994 PMC1226071

[30] Elhawary NA AlJahdali IA Abumansour IS Ezzeldin NE Gaboon N Dandini M Genetic etiology and clinical challenges of phenylketonuria. Hum Genomics. (2022) 16(1):22. 10.1186/s40246-022-00398-9 35854334 PMC9295449

[31] Joint Committee on Infant Hearing. Year 2000 Position Statement: Principles and Guidelines for Early Hearing Detection and Intervention Programs. Available at: http://jcih.org/jcih2000.pdf 10943021

[32] Kemper AR Mahle WT Martin GR Cooley CC Kumar P Morrow WR Strategies for implementing screening for critical congenital heart disease. Pediatrics. (2011) 128(5):e1259–67. 10.1542/peds.2011-1317 21987707

